# Comprehensive 4D-flow cardiac magnetic resonance evaluation of the descending thoracic aorta in aortic regurgitation

**DOI:** 10.1093/ehjimp/qyaf002

**Published:** 2025-01-07

**Authors:** J Urmeneta Ulloa, A Álvarez Vázquez, V Martínez de Vega, L Martínez de Vega, C Andreu-Vázquez, I J Thuissard-Vasallo, M Recio Rodríguez, J A Cabrera

**Affiliations:** Radiology Department, Hospital Universitario Quirónsalud Madrid, Calle Diego de Velázquez, 1, Madrid 28223, Spain; Cardiology Department, Hospital Universitario Quirónsalud Madrid, Calle Diego de Velázquez, 1, Madrid 28223, Spain; Universidad Europea de Madrid, Faculty of Biomedical and Health Sciences, C. Tajo, s/n, 28670 Villaviciosa de Odón, Spain; Radiology Department, Hospital Universitario Quirónsalud Madrid, Calle Diego de Velázquez, 1, Madrid 28223, Spain; Universidad Europea de Madrid, Faculty of Biomedical and Health Sciences, C. Tajo, s/n, 28670 Villaviciosa de Odón, Spain; Radiology Department, Hospital Universitario Quirónsalud Madrid, Calle Diego de Velázquez, 1, Madrid 28223, Spain; Universidad Europea de Madrid, Faculty of Biomedical and Health Sciences, C. Tajo, s/n, 28670 Villaviciosa de Odón, Spain; Radiology Department, Hospital Universitario Ramón y Cajal, M-607, Km. 9, 100, Fuencarral-El Pardo, Madrid 28034, Spain; Universidad Europea de Madrid, Faculty of Biomedical and Health Sciences, C. Tajo, s/n, 28670 Villaviciosa de Odón, Spain; Universidad Europea de Madrid, Faculty of Biomedical and Health Sciences, C. Tajo, s/n, 28670 Villaviciosa de Odón, Spain; Radiology Department, Hospital Universitario Quirónsalud Madrid, Calle Diego de Velázquez, 1, Madrid 28223, Spain; Universidad Europea de Madrid, Faculty of Biomedical and Health Sciences, C. Tajo, s/n, 28670 Villaviciosa de Odón, Spain; Cardiology Department, Hospital Universitario Quirónsalud Madrid, Calle Diego de Velázquez, 1, Madrid 28223, Spain; Universidad Europea de Madrid, Faculty of Biomedical and Health Sciences, C. Tajo, s/n, 28670 Villaviciosa de Odón, Spain

**Keywords:** 4D-flow, cardiac magnetic resonance, aortic regurgitation, descending thoracic aorta, holodiastolic flow reversal

## Abstract

**Aims:**

To assess the reproducibility of 4D-Flow cardiac magnetic resonance (CMR) parameters in the descending thoracic aorta—DTAo—(regurgitant fraction [RF], end-diastolic reverse flow [EDRF], and holodiastolic flow reversal [HDR]), and the relationship with RF in the sinotubular junction (STJ), and the left ventricular end-diastolic volume index (LVEDVI) in patients with chronic aortic regurgitation (AR).

**Methods and results:**

A descriptive study of these variables was conducted. A receiver operating characteristic curve was used to determine the optimal cut-off point. Thirty patients had severe AR (RF ≥ 30%, STJ) and 60 mild-to-moderate (RF < 30%). The mean age was 59 ± 17 years. Left ventricular ejection fraction (LVEF) was 56% (53–61%) and LVEDVI was 94 (76–128) mL/m^2^. Flow in the DTAo at the left inferior pulmonary vein (LIPV) was easily identifiable and measurements were highly reproducible. The intraclass correlation coefficient was 0.969 (95% CI: 0.954–0.980) for RF and 0.929 (95% CI: 0.893–0.952) for EDRF. Flow parameters measured at the LIPV were all significantly greater in the severe AR group: RF (21% vs. 6%, *P* < 0.001), EDRF (20 vs. 4 mL/s; *P* < 0.001), and HDR (20% vs. 8%; *P* < 0.001). Three parameters—presence of HDR, RF ≥ 17%, and EDRF ≥ 7 mL/s at the LIPV—were associated with RF ≥ 30% in the STJ and elevated LVEDVI.

**Conclusion:**

4D-flow CMR can reproducibly assess flow in the DTAo in patients with chronic AR. An RF ≥ 17%, EDRF ≥ 7 mL/s, and/or the presence of HDR in the DTAo (LIPV) were associated with an RF ≥ 30% in STJ and elevated LVEDVI.

## Graphical Abstract

**Graphical abstract:**
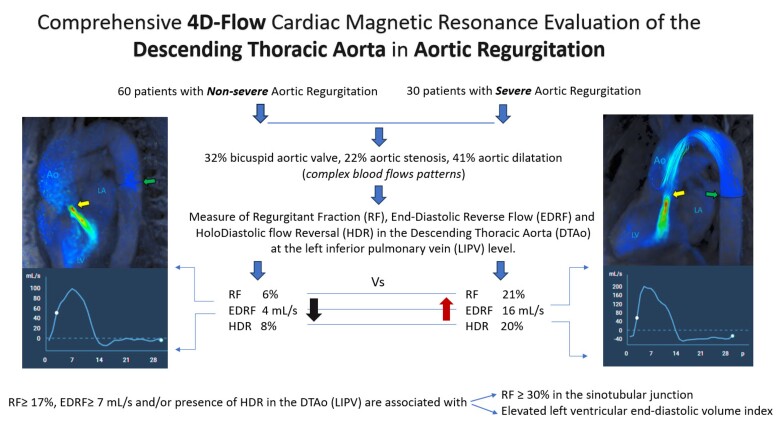

## Introduction

Transthoracic echocardiography (TTE) is widely used to diagnose aortic regurgitation (AR).^1^ TTE is also used to determine the severity of AR. However, in some cases, particularly in patients with moderate-to-severe AR, it can be difficult to precisely determine severity with TTE.^2^ In these cases, patients are commonly referred for cardiac magnetic resonance (CMR) imaging, which can assess quantitative parameters,^2,3^ including regurgitant volume (RV), regurgitant fraction (RF) (two key indicators of AR severity) and flow behaviour in the descending thoracic aorta (DTAo). Moreover, CMR also allows for a more precise—and less operator-dependent—assessment of ventricular volumes than TTE.^3–5^

The 4D-flow sequence is a recent advance in phase-contrast technology. This technique allows for the retrospective evaluation of flow through a single three-dimensional (3D) volume acquisition in any of the three dimensions of space within the acquired volume.^6–8^ 4D-flow CMR can be used to evaluate numerous flow-related parameters relevant to diagnosis, prognosis, and monitoring of the course of disease. The clinical utility of this imaging technique has been demonstrated in a wide range of heart conditions, including congenital, valvular, and aortic disease.^9–15^

Flow assessment in the DTAo is a complementary diagnostic tool for stratifying the severity of AR in TTE. To date, however, only a few studies—all of which used the two-dimensional (2D) technique—have evaluated the use of CMR for this purpose.^16,17^ The 4D-flow technique provides a more comprehensive analysis of blood flow than 2D-CMR and should—at least theoretically—provide a more accurate determination of AR severity. To our knowledge, the role of 4D-flow CMR in assessing flow in the DTAo to aid in grading the severity of AR has not been previously evaluated.

In this context, the main aim of this study was to assess the reproducibility of 4D-flow CMR parameters in the DTAo, specifically RF, end-diastolic reverse flow (EDRF), and holodiastolic flow reversal (HDR). Additionally, we aimed to determine the relationship of these parameters with RF in the sinotubular junction (STJ) and the left ventricular end-diastolic volume index (LVEDVI) in patients with chronic AR. We hypothesized that analysing flow in the DTAo using 4D-flow CMR would provide a more accurate grading of AR severity.

## Methods

### Study population

This was a retrospective study of patients diagnosed with chronic AR—defined as an RF ≥5% at the STJ—who underwent 4D-flow CMR at our institution from 2018 to 2023. Patients were classified into two groups according to AR severity: (i) mild-to-moderate AR (RF > 5% and RF < 30% at the STJ), and (ii) severe AR (RF ≥ 30%^18^ at the STJ).

Exclusion criteria were: (i) age ≤ 18 years, (ii) aortic valve or root replacement, (iii) poor quality CMR imaging studies, and (iv) the presence of cardiomyopathy or other moderate-to-severe valvulopathy as mitral and tricuspid regurgitation. Valve morphology (i.e. bicuspid vs. quadricuspid) and the presence or the absence of aortic/subaortic stenosis or aortic dilation were not considered exclusion criteria.

All participants were treated at our hospital. CMR was performed in accordance with routine clinical practice. This study was approved by the ethics research committee of a third level hospital in our country. All patients completed a questionnaire to provide data on surgical and medical history, allergies, medications, and metal implants (including pacemaker) prior to CMR.

### 4D-flow and CMR acquisition

CMR was performed with the GE Optima MR450w 1.5T (GE Medical Systems; Milwaukee, WI, USA) with a 32-channel surface coil. As part of our standard CMR protocol, we used conventional CMR sequences to assess morphologic and functional parameters (e.g. left ventricle volume, left ventricular ejection fraction—LVEF). Our imaging protocol includes delayed-enhancement images to assess for the presence of ischaemic or non-ischaemic disease. LVEF, ventricular volumes, and anatomical parameters were obtained through routine analysis of white blood CMR sequences. A non-contrast free-breathing isotropic 3D whole-heart MRI was performed before the 4D-flow sequence to assess aortic diameters.

Free-breathing 4D-flow image acquisition was performed simultaneously with the injection of 0.15 mmol/kg of a gadolinium-based contrast agent (Gadovist 1 mmol/mL; Bayer, Mijdrecht, The Netherlands) at a rate of 0.1 mL/s, followed immediately by saline flush. The 3D volume image encompassed the cardiac apex to the aortic arch. The image included the thoracic aorta from the aortic valve to the proximal abdominal aorta, with respiratory compensation and retrospective electrocardiogram-cardiac gating at 30 phases of the cardiac cycle (80%). Acquired resolution was 2.78 × 2.78 × 2.40 mm, with reconstructed resolution of 1.95 × 1.95 × 1.20.

In this cohort, the mean velocity encoding (VENC) parameter was 250 cm/s [range: 250–280], which is adjusted as necessary in cases of valve stenosis. The flip angle was 15°, the repetition time (TR) was 4.25, and the echo time (TE) was 2.25. The total image acquisition time ranges from 7 to 10 min, and the number of views per segment is adjusted automatically based on the heart rate.

### 4D-flow CMR analysis

The raw data were sent for image reconstruction and data correction directly to a cloud software application; Tempus Pixel (Chicago, IL, USA), that provides real-time, 3D post-processing tools to visualize and quantify flows. The flow is colour coded according to blood flow velocity and superimposed on the anatomical data. Two physicians with extensive experience in cardiac imaging (>8 and >11 years, respectively) independently obtained the blood flow parameters using this software.

Flow parameters were determined by contouring the aorta in a double orthogonal plane on the magnitude image and propagating the contours through the cardiac cycle using an automated tracing algorithm tool. Previously, an automatic eddy current condition was performed, with manual adjustments made as necessary to correct for potential offset errors. For the determination of AR severity, the region of interest (ROI) was paced at the STJ in the ascending aorta.^16,18^

We compared perpendicular ROI measurements made at two points in the DTAo: at the pulmonary trunk bifurcation and at the junction of the left inferior pulmonary vein (LIPV) with the left atrium. We measured the RF, EDRF, and HDR at both of these points. The presence of HDR, defined as flow reversal ≥ 10 mL/s persisting through the entire diastole, was established on 4D-flow curves correlated with electrocardiographic gating.^17^

### Statistical analysis

Qualitative variables are reported as absolute (*n*) or relative (%) frequencies, as appropriate. Quantitative variables are reported as means ± standard deviation (SD) if they follow a normal distribution, or as medians with interquartile range (Q1–Q3). The Kolmogorov Smirnov test was used to determine distribution normality.

Chi square tests (or Fisher’s exact tests) were used to compare qualitative variables. Student’s *t*-test or the Mann–Whitney *U*-test was used to compare quantitative variables (parametric or non-parametric, respectively).

To determine the optimal cut-off point of RF in the DTAo to identify patients with severe AR (RF ≥ 30% at STJ), a Receiver Operating Characteristic (ROC) curve was constructed, and the Area Under the Curve (AUC) and its 95% CI were calculated. Sensibility and specificity were calculated for the cut-off value. Similarly, ROC curve was used to determine the optimal cut-off point of EDRF for the diagnosis of severe AR and AUC, sensibility and specificity values were calculated.

The IBM-SPSS statistical software package (SPSS Inc. Chicago, IL, USA) was used to perform the statistical analysis. The significance level alpha was set at 5%.

## Results

### Study population and CMR

The patient demographic and clinical characteristics are shown in *Table 1*.

**Table 1:** Patients’ characteristics, cardiac function, and AR parameters

Variable	Total *n* = 90 (100%)	Non-severe *n* = 60 (66.7%)	Severe *n* = 30 (33.3%)	*P*-Value
Age, years (range)	63 (46–72)	65 (50–73)	59 (44–71)	0.182
Sex				0.225
Female, *n* (%)	22 (24.4)	17 (19)	5 (6)	
Male, *n* (%)	68 (75.6)	43 (48)	25 (28)	
LVEDVI, mL/m^2^	94 (76–128)	79 (72–100)	127 (107–140)	< 0.001
LVESVI, mL/m^2^	42 (31–55)	35 (29–46)	55 (43–67)	< 0.001
LVEF, %	56 (53–61)	57 (53–62)	55 (53–58)	0.162
RF (STJ), %	20 (12–33)	16 (11–20)	36 (33–42)	< 0.001
RF (DTAo, LIPV), %	10 (4–19)	6 (2–11)	21 (17–30)	< 0.001
EDRF (DTAo, LIPV), mL/s	7 (2–16)	4 (1–9)	16 (11–28)	< 0.001
HDR (DTAo, LIPV), *n* (%)	28 (31)	8 (13)	20 (67)	< 0.001
Bicuspid aortic valve, *n* (%)	29 (32)	17 (28)	12 (40)	0.264
Coexisting aortic valve stenosis, *n* (%)	20 (22)	10 (17)	10 (33)	0.073
Dilated ascending aorta ≥45 mm, *n* (%)	11 (12)	9 (15)	2 (7)	0.014
CMR acquisition VENC, cm/s	250 (250–280)	250 (250–270)	250 (250–350)	0.120

All values are given as medians [Q1–Q3] or counts (frequency absolute and relative)

AR, aortic regurgitation; CMR, cardiac magnetic resonance; DTAo,descending thoracic aorta; EDRF, end-diastolic reverse flow; HDR, holodiastolic flow reversal; LIPV, left inferior pulmonary vein; LV, left ventricle; LVEDVI, Left ventricle end-diastolic volume index; LVESVI, Left ventricle end-systolic volume index; LVEF, left ventricle ejection fraction; RF, regurgitant fraction; STJ, sinotubular junction; VENC, velocity encoding.

A total of 90 patients were included. Of these, 60 (67%) had mild-to-moderate (non-severe) AR and 30 (33%) severe AR. The mean (SD) patient age was 59 (±17) years. Most patients were male (*n* = 68; 75.6%).

The median LVEDVI was 94 mL/m^2^ (76–128), the median indexed LV end-systolic volume (LVESVI) was 42 mL/m^2^ (31–55), and the median LVEF was 56% (53–61%).

Valve morphology was bicuspid in 29 cases (32.2%), tricuspid in 60 (67%), and quadricuspid in one (1%). Valvular stenosis was detected in 20 patients and classified as mild in 14% (*n* = 13), moderate in 5.6% (*n* = 5), or severe in 2.2% (*n* = 2). Non-significant subaortic stenosis was present in two patients (2.2%). No clinically moderate-to-severe concomitant valve disease was observed, with mild mitral and tricuspid regurgitation in 6.7% and 2.2% of patients, respectively.

Aortic dilation (aorta ≥ 40 mm) was present in 37 patients (41.1%). Of these, 11 (12.2%) had an aorta diameter ≥ 45 mm and two (2.2%) ≥ 50 mm. Late gadolinium enhancement (LGE) was observed in six (6.7%) patients, all of them with non-ischaemic pattern.

### Interobserver variability by aortic level

*Table 2* shows the 4D-flow CMR measurements made by the two independent operators. Measurements of the RF in the ascending and descending aorta were highly reproducible, as evidenced by the high intraclass correlation coefficient (ICC) values. Standing out among them, RF measurement in the STJ and at the LIPV, with ICC, respectively, 0.960 (95% CI: 0.940–0.974) and 0.969 (95% CI: 0.954–0.980). Assessment of flow in the EDRF was also highly reproducible at the LPIV, with an ICC of 0.929 (95% CI: 0.893–0.952).

**Table 2:** Interobserver correlation coefficient (ICC) between two independent observers

	ICC	95% CI
RF STJ	0.960	0.940–0.974
RF AAo	0.859	0.794–0.905
RF DTAo (LIPV)	0.969	0.954–0.980
EDRF (LIPV)	0.929	0.893–0.952
RF DTAo (PT)	0.965	0.948–0.977

CI, confidence interval; EDRF, end-diastolic reverse flow (LIPV); RF AAo, regurgitant fraction in ascending aorta; RF DTAo (LIPV), regurgitant fraction in descending thoracic aorta (left inferior pulmonary vein); RF DTAo (PT), regurgitant fraction in descending thoracic aorta (pulmonary trunk); RF STJ, regurgitant fraction in sinotubular junction.

### Relationship between flow in the DTAo (RF, EDRF, and HDR), with RF at the STJ, and the LVEDVI

**Figure 1:**
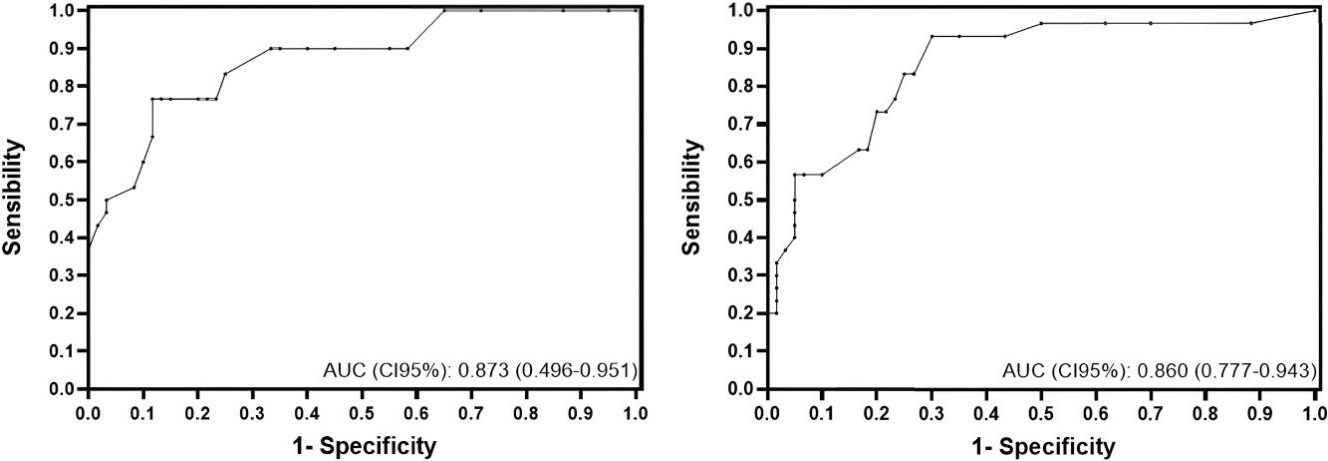
ROC curves. AUC for RF (left) and (EDRF) (right) measured in the DTAo at the LIPV to identify patients with severe AR (RF ≥ 30% at the sinotubular junction).

Based on the ROC curve, the optimal cut-off point for the diagnosis of severe AR (defined as RF ≥ 30% in the STJ) was RF ≥ 17% at the LIPV (sensitivity = 0.77; specificity = 0.88; AUC 95% CI: 0.873 [0.496–0.951]). The optimal cut-off point for EDRF was 7 mL/s (sensitivity = 0.93; specificity = 0.70; AUC 95% CI% 0.860 [0.777–0.943]) (*Figure 1*).

RF values in the STJ ≥ 30% and <30% were significantly correlated with RF (21% and 6%, respectively, *P* ≤ 0.001) and EDRF (16 and 4 mL/s, respectively, *P* ≤ 0.001) values measured in the DTAo at the LIPV.

RF ≥ 30% in the STJ, RF, and EDRF values at the LIPV; ≥17% and ≥7 mL/s, respectively, were significantly associated with a higher LVEDVI and the presence of HDR (*Table 3*). Finally, in the same context, an association was observed between LVEDVI values and the presence of HDR. LVEDVI values were significantly lower in patients without HDR compared with those with HDR (79 [71–103] vs. 127 [101–140] mL/m², respectively; *P* < 0.001).

**Table 3:** Comparison of flow parameters between the STJ (RF) and the DTAo (RF and EDRF) based on the cut-off point for AR severity, in relation to LVEDVI values and the presence of HDR

Variable	RF STJ < 30% *n* = 60	RF STJ ≥ 30% *n* = 30	RF DTAo < 17% *n* = 60	RF DTAo ≥ 17% *n* = 30	EDRF DTAo < 7 mL/s *n* = 44	EDRF DTAo ≥ 7 mL/s *n* = 46	*P*-Value
LVEDVI (mL/m^2^)	79 (72–100)	127 (107–140)	79 (72–100)	127 (107–135)	79 (71–98)	111 (88–135)	< 0.001
HDR							< 0.001
No, *n* (%)	52 (87)	10 (33)	56 (93)	6 (20)	44 (100)	18 (39)	
Yes, *n* (%)	8 (13)	20 (67)	4 (7)	24 (80)	0 (0.0)	28 (61)	

Values are listed as medians [Q1–Q3] or counts (absolute and relative frequency).

EDRF DTAo, end-diastolic reverse flow in descending thoracic aorta; HDR, holodiastolic flow reversal; LVEDVI, left ventricle end-diastolic volume index; RF DTAo, regurgitant fraction in descending thoracic aorta.

## Discussion

The main aim of this study was to assess the reproducibility of 4D-flow CMR parameters in the DTAo (RF, EDRF, and HDR), and to evaluate the relationship between these parameters and RF in the STJ, as well as the LVEDVI in patients with chronic AR. We establish LIPV as a reference point in DTAo where flow measurements are highly reproducible and easily identifiable using 4D-flow CMR. Moreover, the variables (RF, EDRF, HDR) measured at this point were associated with the RF at the STJ and with the LVEDVI. More specifically, we found that an RF ≥ 17%, EDRF ≥ 7 mL/s, and the presence of HDR measured in the DTAo (LIPV) were indicative of severe AR, a finding that confirms the utility of 4D-flow CMR as a complementary diagnostic tool in improving the accuracy of AR grading.

CMR allows for the precise quantification of AR using phase-contrast velocity mapping sequences^19,20^. Myerson *et al*.^21^ found that RF values ≥ 33% (vs. < 33%), and elevated LVEDVI, were associated with progression to surgery and lower survival rates in asymptomatic patients with echocardiographic moderate-to-severe AR. Similarly, CMR-derived volume quantification correlates with AR severity.^22^

Alvarez *et al*.^23^ compared 4D-flow CMR to conventional 2D phase-contrast (PC) CMR in patients with AR, finding that 4D-flow CMR was non-inferior to 2D-PC CMR. One of the key limitations of 2D-PC CMR for quantifying flow at the STJ—an area that has shown high reproducibility^16,24^—is its assumption of laminar flow, which is not always present. For example, in conditions such as bicuspid aortic valve (BAV), valve stenosis, and/or aortic dilation,^25^ flow turbulence near the valve can cause intravoxel dephasing and signal loss during high systolic flow.^24^ The complex blood flow patterns, including regions of swirling and rotational flow, in patients with a dilated (≥40 mm) ascending aorta and a bicuspid^26,27^ or tricuspid^28^ aortic valve can result in inaccuracies when quantifying flow using 2D-PC CMR. In patients with AR, these inaccuracies may lead to variability in the RF quantification of the ascending aorta, particularly when using a single 2D-PC measurement without accounting for regions affected by aliasing. In such cases, even minor variations in positioning at the aortic root or ascending aorta can lead to significant discrepancies in the obtained values.^8^

**Figure 2:**
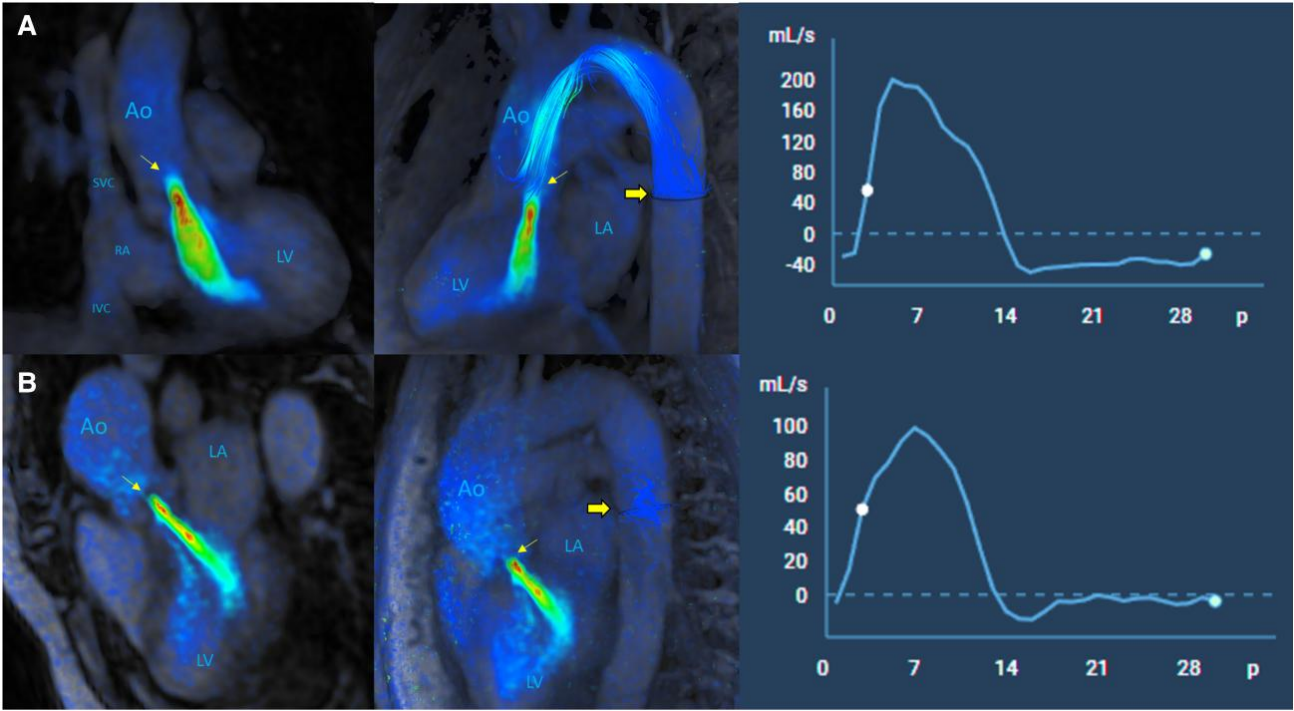
4D-flow CMR evaluation of the DTAo in patients with AR. (*A*) Severe AR: Significant regurgitant flow through the aortic valve, with an RF of 35% at the STJ (thin arrow). Qualitative evidence of HDR in the DTAo is observed using streamlines in 3D volumetric reconstruction (thick arrow) and in the derived curve (upper right, HDR compared with baseline). RF is 32%, and EDRF is 29 mL/s at the LIPV. (*B*) Non-severe AR: Significant regurgitant flow through the aortic valve, with an RF of 30% at the STJ (thin arrow). Qualitative evidence of non-significant flow reversal in the DTAo is observed using streamlines in 3D volumetric reconstruction (thick arrow) and in the derived curve (lower right, without HDR). This is in a patient with AR and complex blood flow patterns (BAV and dilated ascending aorta). RF is 4%, and EDRF is 4 mL/s in the DTAo (LIPV). Abbreviations: Ao, Aorta; AR, aortic regurgitation; DTAo, descending thoracic aorta; EDRF, end-diastolic reverse flow; HDR, holodiastolic flow reversal; IVC, inferior vena cava; LA, left atrium; LIPV, left inferior pulmonary vein; LV, left ventricle; RA, right atrium; RF, regurgitant fraction; STJ, sinotubular junction; SVC, superior vena cava.

In routine clinical practice, TTE is the primary tool used to assess flow behaviour in the DTAo and to determine the presence or the absence of HDR and end-diastolic velocity (EDV)^29,30^. To date, only a few studies have described the use of 2D-CMR for this purpose. In the present study, we used 4D-flow CMR to analyse flow parameters in the DTAo, including RF and EDRF (which is similar to EDV in TTE).^31^ As our results show, reproducibility was excellent when measuring these parameters at the LIPV, an easily identifiable region that is likely less influenced by flow turbulence than the more proximal regions of the DTAo or those close to the distal aortic arch.^29^ Our findings indicate that the analysis of 4D-flow CMR at the level of the DTAo can aid in grading the severity of AR, particularly in cases with complex blood flow patterns (*Figure 2*, Supplementary data online, *Videos S1* and *S2*), consistent with previous studies that have used 2D-CMR.^16,17^

Kammerlander *et al*.^16^ used 2D-PC CMR to assess flow in the DTAo in 232 patients with AR (44 with severe AR), finding that the presence of HDR was significantly associated with larger ventricular volumes, lower LVEF values, and higher RF. Similarly, Bolen *et al*.^17^ used 2D-PC CMR to evaluate flow in the DTAo in 18 patients with moderate-to-severe AR, demonstrating that HDR presence was a strong predictor of severe AR (sensitivity, 100%; specificity, 93%). As noted by those authors, and corroborated by our group in this study using 4D-flow CMR, this assessment proves particularly useful in patients with complex blood flow patterns in the ascending thoracic aorta. These findings suggest that HDR is an independent indicator of AR severity that complements CMR-based RF quantification.

To our knowledge, this study is the first to demonstrate the value of 4D-flow CMR for assessing blood flow in the DTAo in patients with AR. Compared with 2D-PC CMR, the 4D technique ensures flow perpendicularity, enabling a comprehensive analysis of flow throughout the DTAo. We found that flow parameters in the DTAo are associated with the RF in the STJ and LVEDVI. Furthermore, an RF ≥ 17%, an EDRF ≥ 7 mL/s, and/or the presence of HDR in the DTAo (LIPV) are associated with an RF ≥ 30% in the STJ and elevated LVEDVI values. These findings highlight that assessing flow in the DTAo with 4D-flow CMR is a robust and valuable complementary diagnostic tool in the comprehensive evaluation of AR.

In cases where AR severity, as measured by TTE, is uncertain or where RF values in the STJ are incongruent due to the presence of complex blood flow patterns, 4D-flow CMR can provide crucial data to ensure an accurate determination of AR severity. The findings of this novel study could serve as the basis for future prospective studies evaluating the impact of AR in patients with significantly altered flows in the DTAo but RF < 30% in the STJ. In such cases, accurately characterizing flow patterns and AR severity is essential for selecting the optimal therapeutic strategy (e.g. early surgery and/or close surveillance) and for prognostic purposes, helping predict the disease trajectory.

### Limitations

This study has several limitations. First, although complex blood flow patterns in the DTAo have less influence on flow quantification than those in the proximal ascending aorta, previous studies have shown that the presence of non-uniform flow in the DTAo can lead to measurement discrepancies.^32^ Nevertheless, as we have demonstrated, in most cases this seems to have minimal impact on measurements taken in the mid-distal regions of the DTAo (considering the LIPV as the reference point). Another limitation is that, in some patients, we had to increase the VENC due to aortic valve stenosis and BAV. As a result, this parameter was not uniform in the whole cohort. However, previous studies have shown that regurgitant blood flow volume has no clinically-relevant dependency on the VENC level.^33^ The moderate sample size and the lack of healthy controls without valvular heart disease can also be considered limitations. Temporal resolution could be considered a theoretical limitation of the 4D-flow technique; however, as demonstrated by previous groups,^8^ it is sufficient to obtain reproducible and valid results in clinical practice. Finally, the 4D-flow sequence was performed on a single 1.5T machine (GE 1.5T MRI) and the flow analyses were performed with a specific software tool. Consequently, we cannot ensure the replicability of our findings under different conditions using different MRI machines, field strengths, and/or software.

## Conclusion

Our findings demonstrate that 4D-flow CMR can accurately and reproducibly assess blood flow in the DTAo. These results suggest that the use of 4D-flow CMR could enhance the accuracy of grading AR. An RF ≥ 17%, EDRF of ≥7 mL/s, and/or the presence of HDR in the DTAo (LIPV) were associated with an RF of ≥30% in the STJ and elevated LVEDVI in our study population.

## Supplementary data

Supplementary data are available at *European Heart Journal - Imaging Methods and Practice* online.

## Consent

All participants signed an informed consent form, which included permission to publish anonymized clinical and/or imaging data in scientific or educational reports.

## Supplementary Material

qyaf002_Supplementary_Data

## Data Availability

Data are available from the corresponding author on reasonable request.

## Lead author biography

J. Urmeneta Ulloa is a Cardiologist at Hospital Universitario Quirónsalud, Madrid, Spain. MD, PhD (Cum Laude and Extraordinary Award). He is an expert in advanced cardiac imaging techniques, EACVI-certified in CMR, CT, and TTE, and accredited as an Expert in Advanced Echocardiography by the Spanish Society of Cardiology. He holds four master’s degrees and four expert-level courses in the field of cardiac imaging and an author of 25 PubMed-indexed publications as first author.
