# Supplementary material for: Comprehensive 4D-flow cardiac magnetic resonance evaluation of the descending thoracic aorta in aortic regurgitation
Source: Eur Heart J Imaging Methods Pract. 2025 Jan 7;3(1):qyaf002. doi: 10.1093/ehjimp/qyaf002 (PMC11758371; doi:10.1093/ehjimp/qyaf002)
Supplement: qyaf002_Supplementary_Data [file qyaf002_supplementary_data.zip › Videocaption.docx]

**Video 1.** 4D-Flow CMR evaluation of the descending thoracic aorta (DTAo) in a patient with aortic regurgitation (AR). Qualitative evidence of holodiastolic flow reversal (HDR) is observed using streamlines in a patient with severe AR.

**Video 2**. 4D-Flow CMR evaluation of the descending thoracic aorta (DTAo) in a patient with aortic regurgitation (AR). Qualitative evidence of non-significant flow reversal is observed using streamlines in a patient with moderate AR and complex blood flow patterns in the ascending aorta.
